# Supplementary material for: Effect of seedling size on post-planting growth and survival of five Mexican Pinus species and their hybrids
Source: PeerJ. 2024 Dec 20;12:e18725. doi: 10.7717/peerj.18725 (PMC11665424; doi:10.7717/peerj.18725)
Supplement: Supplemental Information 1 [file peerj-12-18725-s001.docx]

**Supplementary Table 1**

Number of seedlings (*N*) of the five pure (-P) *Pinus* species and their seed provenances in the study, in each separate field trial and in both trials together, 15 months after sowing in the nursery

| **Group** | **Provenances** | ***N***  **Mesa Alta** | ***N***  **Mesa Seca** | ***N***  **Both trials together** |
| --- | --- | --- | --- | --- |
| **PA-P** | **PA C** | 4 | 1 | 5 |
|  | **PA GI** | 22 | 1 | 23 |
| **PD-P** | **PD A** | 46 | 44 | 90 |
|  | **PD ME** | 1 | 1 | 2 |
|  | **PD P** | 3 | 5 | 8 |
| **PE-P** | **PE M** | 2 | 3 | 5 |
|  | **PE MM** | 29 | 51 | 80 |
|  | **PE MM2** | 32 | 43 | 75 |
|  | **PE MP** | 36 | 50 | 86 |
|  | **PE MP2** | 67 | 78 | 145 |
|  | **PE MP3** | 43 | 56 | 99 |
|  | **PE MP4** | 34 | 55 | 89 |
| **PL-P** | **PL M** | 11 | 13 | 24 |
|  | **PL TS** | 8 | 13 | 21 |
| **PT-P** | **PT AG** | 34 | 42 | 76 |
|  | **PT BA** | 7 | 15 | 22 |
|  | **PT MC** | 10 | 14 | 24 |
|  | **PT O** | 3 | 11 | 14 |
|  | **PT P** | 3 | 6 | 9 |
|  | **PT PA** | 5 | 12 | 17 |
|  | **PT SE** | 1 | 2 | 3 |
|  | **PT TI** | 23 | 25 | 48 |
| **Total numbers** |  | **424** | **541** | **965** |

Note: PA‐P = *P. arizonica*, PD‐P = *P. durangensis*, PE‐P = *P. engelmannii*, PL‐P = *P. leiophylla* and PT‐P = *P. teocote* (see details of seed provenances in Hernández-Velasco et al. 2021).
