# Supplementary material for: Effect of seedling size on post-planting growth and survival of five Mexican Pinus species and their hybrids
Source: PeerJ. 2024 Dec 20;12:e18725. doi: 10.7717/peerj.18725 (PMC11665424; doi:10.7717/peerj.18725)
Supplement: Supplemental Information 2 [file peerj-12-18725-s002.docx]

**Supplementary Table 2**

Number (*N*) of hybrid seedlings (-H) of the five *Pinus* species and their seed provenances in the study, in each separate field trial and in both trials together, 15 months after sowing in the nursery

| **Group** | **Provenances** | ***N***  **Mesa Alta** | ***N***  **Mesa Seca** | ***N***  **Both trials together** |
| --- | --- | --- | --- | --- |
| **PA-H** | **PA C** | 2 |  | 2 |
|  | **PA GI** | 10 | 1 | 11 |
| **PD-H** | **PD A** | 11 | 16 | 27 |
|  | **PD ME** | 33 | 80 | 113 |
|  | **PD P** | 5 | 7 | 12 |
| **PE-H** | **PE M** | 1 | 3 | 4 |
|  | **PE MM** | 50 | 94 | 144 |
|  | **PE MM2** | 25 | 29 | 54 |
|  | **PE MP** | 26 | 30 | 56 |
|  | **PE MP2** | 21 | 38 | 59 |
|  | **PE MP3** | 43 | 45 | 88 |
|  | **PE MP4** | 45 | 50 | 95 |
| **PL-H** | **PL M** | 20 | 38 | 58 |
|  | **PL TS** | 2 | 5 | 7 |
| **PT-H** | **PT AG** | 22 | 35 | 57 |
|  | **PT BA** | 5 | 5 | 10 |
|  | **PT CR** | 17 | 37 | 54 |
|  | **PT MC** | 5 | 1 | 6 |
|  | **PT O** | 26 | 23 | 49 |
|  | **PT P** |  | 2 | 2 |
|  | **PT PA** | 16 | 15 | 31 |
|  | **PT SE** | 31 | 44 | 75 |
|  | **PT TI** | 15 | 13 | 28 |
| **Total numbers** |  | **431** | **611** | **1,042** |

Note: PA‐H = hybrids of *Pinus arizonica* × *P. durangensis* genetically more similar to *P. arizonica*; PD‐H = hybrids of *P*. *durangensis* × *P*. *arizonica* genetically more similar to *P*. *durangensis* and *P. durangensis* × *P. engelmannii* genetically more similar to *P. durangensis*; PE‐H = hybrids of *P. engelmannii* × *P. arizonica* genetically more similar to *P*. *engelmannii*; PL‐H = hybrids of *P. leiophylla* × *P. teocote* genetically more similar to *P. leiophylla*; PT‐H = *P*. *leiophylla* × *P. teocote* genetically more similar to *P. teocote* (see details of seed provenances in Hernández-Velasco et al. 2021).
