# Supplementary material for: Effect of seedling size on post-planting growth and survival of five Mexican Pinus species and their hybrids
Source: PeerJ. 2024 Dec 20;12:e18725. doi: 10.7717/peerj.18725 (PMC11665424; doi:10.7717/peerj.18725)
Supplement: Supplemental Information 3 — Significant correlation (rs) between the root collar diameter (RCD, mm), height (H, cm), robustness index (HRCD, cm/mm2) of seedlings before planting in 2018 and RCD3, H3 and the survival rate (SR) of the same seedlings 44 months after planting in the field trials, for each pine pure species (−P) and their hybrids (−H), and their 95% confidence interval (CI95%) by bootstrap (1,000,000 iterations). [file peerj-12-18725-s003.docx]

**Table S3** Significant correlation (*r_s_*) between the root collar diameter (*RCD*, mm), height (*H*, cm), robustness index (*HRCD*, cm/mm^2^) of seedlings before planting in 2018 and *RCD_3_*, *H_3_* and the survival rate (*SR*) of the same seedlings 44 months after planting in the field trials, for each pine pure species (-P) and their hybrids (-H)

| **Group** | ***Var*** | ***r_s_*[*Var* vs *RCD_3_*] (*CI_95%_*)** | ***r_s_*[*Var* vs *H_3_*] (*CI_95%_*)** | ***r_s_*[*Var* vs *SR*] (*CI_95%_*)** |
| --- | --- | --- | --- | --- |
| **PL-P** | *RCD* | 0.79**(0.64 - 1.0) | 0.49*(0.19 - 1.0) | -0.16 |
| **PL-H** | *RCD* | 0.51*(0.17 - 0.95) | 0.17 | 0.14 |
| **PT-H** | *RCD* | 0.23**(0.08 - 0.38) | 0.13 | -0.11 |
| **PE-H** | *RCD* | 0.22***(0.10 - 0.33) | 0.10 | 0.02 |
| **PE-P** | *RCD* | 0.20***(0.09 - 0.32) | 0.08 | 0.08 |
| **PT-P** | *RCD* | 0.15 | 0.01 | -0.21*(-0.35 - -0.09) |
| **all** | *RCD* | 0.29***(0.23- 0.37) |  | -0.02 |
| **PL-H** | *H* | 0.40**(0.15 - 0.80) | 0.65***(0.44 - 1.0) | 0.40**(0.20 - 0.64) |
| **PE-H** | *H* | -0.11 | 0.29***(0.17 - 0.41) | 0.04 |
| **PE-P** | *H* | 0.02 | 0.24***(0.12 - 0.36) | 0.03 |
| **PD-H** | *H* | 0.27 | 0.20 | 0.24*(0.08 - 0.40) |
| **PT-H** | *H* | -0.12 | 0.08 | 0.21***(0.09 - 0.32) |
| **PT-P** | *H* | -0.10 | 0.17 | 0.20**(0.07- 0.32) |
| **all** | *H* |  | 0.40***(0.34 - 0.46) | 0.10***(0.04- 0.14) |
| **PT-H** | *HRCD* | -0.24**(-0.40 - -0.09) | -0.01 | 0.19*(0.08 - 0.29) |
| **PE-H** | *HRCD* | -0.22***(-0.34- - 0.12) | 0.03 | 0.09 |
| **PE-P** | *HRCD* | -0.18**(-0.30 - -0.07) | 0.09 | -0.05 |
| **PT-P** | *HRCD* | -0.16 | 0.04 | 0.26**(0.14 - 0.41) |
| **all** | *HRCD* |  |  | 0.06**(0.02 - 0.12) |

Note: var = variable, *CI_95%_* = 95% confidence interval by 1,000,000 bootstrap iterations and subsamples size of 50 seedlings, *RCD_3_* = root collar diameter (mm) 44 months after planting in the field trials, *H_3_* = height (cm) after 44 months of planting in the trials; PA‐P = *Pinus arizonica* pure species, PD‐P = *P. durangensis* pure species, PE‐P = *P. engelmannii* pure species, PL‐P = *P. leiophylla* pure species and PT‐P = *P. teocote* pure species; PA‐H = hybrids of *Pinus arizonica* × *P. durangensis* genetically more similar to *P. arizonica*; PD‐H = hybrids of *P*. *durangensis* × *P*. *arizonica* genetically more similar to *P*. *durangensis* and *P. durangensis* × *P. engelmannii* genetically more similar to *P. durangensis*; PE‐H = hybrids of *P. engelmannii* × *P. arizonica* genetically more similar to *P*. *engelmannii*; PL‐H = hybrids of *P. leiophylla* × *P. teocote* genetically more similar to *P. leiophylla*; PT‐H = *P*. *leiophylla* × *P. teocote* genetically more similar to *P. teocote;* all = across all species and their hybrids (see more in Hernández-Velasco et al. 2021; Sanchez-Hernandez et al. 2022).
